# Supplementary material for: A developmental single-cell atlas of the Drosophila visual system glia reveals cell type diversification and subcellular mRNA compartmentalization
Source: PLoS Biol. 2026 Sep 28;24(9):e3004015. doi: 10.1371/journal.pbio.3004015 (PMC13649123; doi:10.1371/journal.pbio.3004015)
Supplement: Supplementary file 1 [file pbio.3004015.s020.docx]

Response to Reviewers:

**Reviewer 1:**

1. About the origin of medulla neuropil glia, is it known how many neuropil glia are made from a single medulla neuroblast? If more than one, how about the glia diversity from a single medulla neuroblast?

The current consensus is that medulla neuropil glia (mng) are generated during the last temporal window of competency of medulla Neuroblasts (NB) (Li et al., 2013, ref. 56; Zhu et al., 2022, ref. 57; Konstantinides et al., 2022, ref. 25). However, we do not know whether the NB terminally divides symmetrically to produce two glial cells or asymmetrically producing only one. Unpublished work from our lab studying single NB clones strongly suggests that there is only one mng generated from a single NB. This favors the model we propose in the manuscript that ALG vs EG fates are specified long after the birth and migration of mng. Our transcriptomic data indicate a unique mng trajectory in mng pseudotime alignment (**Fig. 5A-D**) and lineage tracing data (both EG and ALG neuropil glia, including all morphotypes, are generated from L3 mng, **Fig. 4D, G**) strongly suggest that a homogeneous mng population diversifies later during development. We have directly tested the alternative hypothesis: due to the salt-and-pepper expression of *Hand* in L3 mng (Fig. 4E), we hypothesized that this TF may be an early marker of EG *vs.* ALG fate. However, lineage tracing experiments with *Hand-T2A-*Gal4 showed that both EG and ALG neuropil glia were observed in the adult (**Fig. 4H** and **S2C**).

2. It is unclear about the nature of lineage tracing on Hand-expressing medulla neuropil glia. Any evidence of cell proliferation?

To address the adult identity of larval *Hand-T2A*-Gal4 mng expressing medulla neuropil glia, lineage tracing experiments were performed in two ways:
- First, we used an *Act5C>STOP>nuc-lacZ* memory cassette (Struhl and Basler 1993, ref. 58) combined with *Hand-T2A*-Gal4>>UAS-*flp*. In this experiment, *nuc-lacZ* in adult brains is only expressed in cells that have expressed *Hand-T2A*-Gal4 at L3 (**Fig. S2C**). From this experiment, we observed that *Hand-T2A-*expressing glial cells are localized around the medulla neuropil in the adult brain, consistent with a broad mng identity including the different known mng morphotypes (proximal, distal and lateral).
- Second, to further explore the morphology of *Hand-T2A*-expressing mng glia, we used a FLEXAMP memory cassette (Bertet et al., 2014, ref. 55). In this case, Flp-induced recombination causes the expression of LexA>>LexAop-*myrGFP*. This experiment allowed us to observe that all adult mng morphologies, including EG and ALG and all their morphotypes, were generated by cells that expressed *Hand-T2A*-Gal4 during L3 (**Fig. 4H**). These experimental procedures are described in detail in the **Methods** section under the title "**Validation of glial trajectories**".

We have not found any evidence of cell proliferation among L3 mng, as shown by PH3 staining (see attached figure). Proliferative activity can be seen in eg/mg glia, as described in Chen et al., 2016 (ref. 45), but not in mng.

3. Please comment if similar phenomena of glia fate diversification and plasticity are observed in other systems and model organisms.

It has been previously reported that, in the central brain of *Drosophila* ensheathing and astrocyte-like glia also come from a common progenitor (Omoto et al., 2015, ref. 19; Ren et al., 2018, ref. 78; Kato et al., 2020, ref. 79). Together with our own observations for medulla and lamina neuropil glia, the plasticity of neuropil glia progenitors to produce ALG and EG types seems to be a general theme in *Drosophila* development. We thank the reviewer for highlighting this point, and we have included a paragraph on this topic in the Discussion.

Regarding the comparison with other models, we prefer to be cautious in drawing comparisons with vertebrates because, even if glial function or morphology can appear similar, this is likely due to convergent evolution rather than any significant developmental similarity. For example, a recent single cell study of human cortex development has shown that oligodendrocytes and astrocytes are generated from the same Radial Glial cells that are neuroglioblasts, like *Drosophila* optic lobe NBs (Ding et al., 2026, PMID: 41565813). Although it is tempting to establish comparisons, the diversification of some glial types during development is well supported for *Drosophila* neuropil glia, but we couldn't find direct evidence that this is the case in other models.

**Reviewer 2:**

This is an impressive and important study that leverages existing scRNAseq datasets to delineate optic lobe glial developmental trajectories, subtypes, and intriguing subcellular differences between soma and processes. The analyses here will open new avenues for studying and manipulating fly glia, as well as for separating soma from cellular processes in scRNAseq datasets.

I support publication of this study with no additional experiments required, but with some minor and major suggestions to make the MS more broadly readable outside of the fly field.

**Major comments:**

1) Fig1: before diving into approach, a schematic of the fly glia here (even if similar to take-home messages in Fig7) would be very useful for non-experts

We agree with the reviewer that more context about the optic lobe anatomy would be helpful for the non-expert reader. Therefore, we have reorganized the Figure for increased clarity and added a schematic representation of the larval and adult brains that are displayed in the immunostainings in **Fig.1**.

2) Fig6 is REALLY important. I would highly encourage authors to put schema of the problem that they identified (cell body vs processes) at top of figure. Authors are encouraged to show top 10 DEGs for PC vs CB, for both cortex glia and chiasm glia? (or show GO analysis, but DEGs maybe more useful). I am wondering if the graphed gene examples (ie fabp) are the most differentially expressed, or were selected for other reasons such as historical precedent for process enrichment

We very much value the reviewer's comment about **Fig. 6**. We have included a schematic representation of the main problem we found, *i.e.* the fact that cells with large processes can be broken up during dissociation steps and result in mRNA containing fragments with and without nuclei (see new **Fig. 6D**).
The representative genes with higher expression in cell processes as compared to the cell body (*fabp*, *Act5C* and *FK506-bp2*) were not selected as the most differentially expressed. The full list of differentially expressed genes in the Cortex_PC and Chiasm_PC clusters is shown in **Table S3**. As suggested by the reviewer, we have considered showing the list of the top10 DEGs for these clusters in the figure, but we find that that data can be misleading if presented as such in the main figure: Seurat ranks DEGs by *adjusted p-value*, which heavily favors genes that have a significant difference in the presence/absence between the compared clusters (*i.e.*, a gene that is expressed in 90% of cells in CB and only 20% of cells in PC will score highly). Given the relative low abundance of mRNA counts in PC clusters, there is no single gene with a % expressed that is higher in PC than in CB either in Cortex or in Chiasm glia. This is the case for *fabp*, *Act5C* and *FK506-bp2*, which present a low ranking based on *adjusted p-value*, but still present higher expression in PC vs CB (**Fig. 6K, L, O, P**). The top10 DEGs in Cortex glia are shown below (pct.1 refers to percent expressed in Cortex_PC, and pct.2 to percent expressed in Cortex_CB):

**top10 DEGs Cortex_PC**

| # |  | p_val | avg_log2FC | pct.1 | pct.2 | p_val_adj |
| --- | --- | --- | --- | --- | --- | --- |
| 1 | Wdr82 | 2.04E-20 | 0.28243976 | 0.078 | 0.468 | 2.19E-16 |
| 2 | CG43658 | 1.91E-18 | 0.25772128 | 0.053 | 0.355 | 2.05E-14 |
| 3 | SmD1 | 2.58E-18 | 0.28013188 | 0.153 | 0.677 | 2.76E-14 |
| 4 | CG7787 | 2.06E-17 | 0.26299737 | 0.06 | 0.371 | 2.21E-13 |
| 5 | l(3)neo38 | 6.76E-17 | 0.28453298 | 0.139 | 0.613 | 7.26E-13 |
| 6 | Caf1 | 7.25E-17 | 0.25053961 | 0.15 | 0.645 | 7.79E-13 |
| 7 | CG9099 | 5.13E-15 | 0.25432297 | 0.177 | 0.694 | 5.50E-11 |
| 8 | Art1 | 5.16E-15 | 0.40278169 | 0.098 | 0.468 | 5.54E-11 |
| 9 | CG4646 | 5.36E-15 | 0.29340295 | 0.094 | 0.452 | 5.75E-11 |
| 10 | CG17776 | 1.18E-14 | 0.27327092 | 0.185 | 0.71 | 1.26E-10 |

**Tested genes in Cortex_PC**

| 88 | fabp | 4.16E-10 | 0.4121565 | 1 | 1 | 4.46E-06 |
| --- | --- | --- | --- | --- | --- | --- |
| 259 | FK506-bp2 | 4.27E-05 | 0.66062779 | 0.788 | 0.952 | 0.45801439 |

*The top10 DEGs in Chiasm glia are shown below (pct.1 refers to percent expressed in Chiasm_PC, and pct.2 to percent expressed in Chiasm_CB):*

**top10 DEGs Chiasm_PC**

| # | gene | p_val | avg_log2FC | pct.1 | pct.2 | p_val_adj |
| --- | --- | --- | --- | --- | --- | --- |
| 1 | RhoGAP19D | 3.42E-25 | 0.25623873 | 0.204 | 0.883 | 3.67E-21 |
| 2 | mRpL46 | 1.75E-24 | 0.2816496 | 0.134 | 0.697 | 1.88E-20 |
| 3 | CG14229 | 1.03E-22 | 0.31338393 | 0.11 | 0.607 | 1.10E-18 |
| 4 | MED31 | 4.00E-22 | 0.29140463 | 0.146 | 0.697 | 4.29E-18 |
| 5 | DCP1 | 4.10E-21 | 0.2600626 | 0.131 | 0.641 | 4.40E-17 |
| 6 | Rae1 | 1.62E-20 | 0.26351664 | 0.177 | 0.752 | 1.74E-16 |
| 7 | mRpL38 | 2.46E-20 | 0.27153185 | 0.101 | 0.552 | 2.64E-16 |
| 8 | bc10 | 7.56E-20 | 0.27585156 | 0.088 | 0.51 | 8.11E-16 |
| 9 | mip40 | 8.79E-20 | 0.27156076 | 0.155 | 0.676 | 9.44E-16 |
| 10 | CG5708 | 1.12E-19 | 0.26020547 | 0.165 | 0.703 | 1.20E-15 |

**Tested genes in Chiasm_PC**

| 119 | Act5C | 1.21E-07 | 0.30512897 | 1 | 1 | 0.00130325 |
| --- | --- | --- | --- | --- | --- | --- |
| 121 | FK506-bp2 | 2.75E-07 | 0.3811199 | 0.948 | 0.986 | 0.00295272 |

Therefore, we indicated in the text that *fabp*, *Act5C* and *FK506-bp2* were selected based on a literature search that pointed that orthologs of these genes have been shown to have mRNA subcellular localization, which made them great candidates for our analysis. In **Fig. 6** we show that indeed, mRNAs of these genes are expressed at higher levels in clusters of Processes compared to their Cell Bodies, despite them not being ranked at the top of differentially expressed genes.
Similarly, the application of GO term analysis is directly dependent on the discovery of relevant genes enriched in PC clusters: if the gene list is flawed (i.e., the problems aforementioned for the list of DEG genes in PCs vs. CBs), the GO annotation would not be informative. A better method than DEG analysis should be designed to identify process-enriched genes, but this is beyond the scope of this project in which we focus on the validation of our initial observation.

3) Do the authors have any examples of interpretations that were changed upon excluding processes compared to soma? Are developmental trajectories altered by the inclusion of these processes? (is important information contained exclusively in these processes that is lost if those clusters are excluded from the bulk of the analyses in this data?).

We thank the reviewer for raising this point. Indeed, PC clusters have lower UMI counts and present lower abundance of TF genes, features that can affect trajectory inference and pseudotime alignment. However, upon careful examination of our data, we do not think that PC clusters impacted our trajectory alignments for several reasons: 1) The trajectory alignment (and the pseudotime inference, in the case of mng) were performed combining either medulla or lamina neuropil glia cells annotated from all the time points from the optic lobe atlases (L3, P15, P30, P50, P70 and Adult), while we only annotated PC vs CB clusters in the L3 and Adult datasets (see reply to reviewer's *Minor Comment 2* below).
2) The generation of PC clusters due to cell fragmentation is more dramatic in glial cells of a very large size, such as Chiasm and Cortex glia in the L3 dataset (**Fig. S5**). However, not all glial clusters are split: out of all the medulla and lamina neuropil glia clusters, only Medulla_EG (Adult) was detected as a PC cluster by our approach (**Fig. S6B, C**). 3) In the case of cell types that do split in PC and CB, L3 Cortex glia does not change transcriptionally through pupal development, while Chiasm glia follows a linear trajectory of gradual change (**Fig. 4A**), so the presence or absence of PC clusters would not be relevant in our interpretation of their trajectories. 4) Importantly, the bifurcation event between medulla ALG and EG fates occur around P30 (**Fig. 5C**), a developmental time where we do not have identified PC clusters.
We do agree that PC clusters are a relevant issue in the inference of developmental trajectories for the predicted lobula neuropil glia, since most of the clusters identified as lobula neuropil glia ( Astrocytes_1, 2, 4 and part of Ensheathing_1) were identified as containing processes (**Fig. S6C**). However, all our analyses and conclusions in this manuscript regarding lobula neuropil glia are speculative due to the limited quality of these clusters. Therefore, we have tried to be very explicit in our Results and Discussion about the high level of uncertainty we have towards these cell types.

4) The authors propose a pipeline for identifying CB vs PC. Does this work in non-fly or different labs' data? Attempting the pipeline on other organism, or commenting on if this problem exists in other datasets, would be greatly appreciated by the broader community! Would a similar problem exist for neuronal soma vs processes?

We agree with the reviewer that expanding the use of our PC vs CB pipeline to other datasets would increase the appeal to the community. In fact, we have analyzed datasets with:
*a)* the neuronal clusters of the adult *Drosophila* optic lobe (Özel et al., 2021, ref. 24) and
*b)* a macaque retina dataset that contains Müller glia and photoreceptors, both of which are very large cells that might form processes' fragments upon dissociation (Peng et al., 2019, PMID: 30712875).
In optic lobe neurons, we have been able to find PC clusters for 14 of 71 analyzed neuronal types using the same pipeline designed for glia (Approach 2 in **Fig. S6A**). In the case of the mammalian retina, we have also been able to identify PC clusters for both Müller glia and photoreceptors using our detection approach. However, although these analyses are promising, they are yet at a very preliminary stage and would require extensive work for their validation. The systematic analysis of fragmentation and mRNA localization in different datasets is beyond the scope of this work and deserves a dedicated study.

a. For example, in PMID 38839958, FigS1f, elav and nSyb reads appear quite high in repo+ glia sorted by FACS from repo-GAL4 > TRE-dsRed,UAS-GFP brains. Author logic is that neurons are dsRednegGFPneg, AP1neg glia are dsRednegGFP + , and AP1+ glia are dsRed+GFP +. Wondering if the soma vs PC is driving some of this messiness.

We would like to note that, in this specific example, the dataset they analyze is obtained through FACS-sorting and bulk RNAseq of the cellular populations of interest. Crucially, our PC vs CB detection approach relies on single-cell resolution metrics, such as UMI content or cluster similarity, and therefore our method cannot be directly applied to bulk RNAseq datasets which represent the average expression of pooled populations of cells.
We speculate that one of the reasons driving the high levels of nSyb in glial cells in this study may be cell contamination with neurons or neuron's debris during the FACS sorting process of the glial cells, which is common when isolating low abundance cell types by FACS.

5) Kato et al (PMID 32051172) claim that "larval EG dedifferentiate, proliferate and redifferentiate into adult ALG and EG.", based on analysis withGs2+ and Naz+ IHC experiments in the central brain. Similarities between ALG and EG subtypes is also suggested by this paper, with "neuropil glia that transcriptionally appears as a single cell class in larvae, splits into ensheathing (EG) and astrocyte-like (ALG) glia types during pupal stages." If possible, authors may comment on these different outcomes (noting also optic lobe versus central brain differences) in the discussion.

We thank the reviewer for bringing up this relevant point that we missed in our discussion. Indeed, there is a similarity in that both adult ALG and EG neuropil glia can be generated from individual pools of progenitors. The main difference is that, in the case of the Central Brain (Kato et al., 2020 results), the progenitors can be either undifferentiated gcm+ cells or mature larval EG that had de-differentiated. We think that this is consistent with our observation (that mng fate is initially not committed to either ALG or EG and that this distinction is set later during development) and therefore strengthen our discussion.
We have modified the discussion to include this view, together with a broader discussion of neuropil glia diversification in *Drosophila* (see response to reviewer 1 above).

**Minor comments:**

1) "Cortex glia is" and similar examples of pluralities of glia given singular verbs exist in the MS; I encourage authors to make sure collective groupings of glia are given plural verbs!

a. Ie "neuropil glia that transcriptionally appears as a single cell class in larvae, splits into ensheathing (EG) and astrocyte-like (ALG) glia types during pupal stages."

We agree with the reviewer and have corrected the error in the main text: in this case we are referring to both lamina and medulla neuropil glia cell types, so the plural form of the verb should be used. As a general rule, since "glia" can be used as the singular or plural form of the noun, we have tried to be consistent throughout the text by using a plural verbal form when referring to groups of glial cells and the singular form when referring to a glial cell type. We have reviewed our manuscript again, and we apologize if any error is still present.

2) Digestibility of all figures would be improved by having the life stage noted within the figure panel rather than only fig legend (ie Fig. 1D as L3 and Fig. 1E as adult).

We agree with the reviewer and have updated **Fig. 1**, where this was not explicitly mentioned, to clearly label the life stages.

3) Authors mention that mbp translation is induced by activity; is there any relationship between electrically active brains (>P50) and the types or abundances of cortex glial process transcriptomes?

We appreciate the reviewer's comment and agree that this is an interesting path to explore. In this study, we have limited our classification of Processes vs Cell Body clusters to our L3 Larva and Adult datasets. Importantly, what initially led to the identification of PC vs CB clusters was the observation that cells with very similar transcriptomic profile were still consistently classified as separate clusters (Fig. S5B). For this to happen, the dataset needs to be relatively large, as it is the case for our L3 and Adult atlases. However, for intermediate time points (P15, P30, P50 and P70), this was not the case. Within the scope of this work, we expect that the differences observed in L3 PCs and Adult PCs are mainly driven by the transcriptional changes associated with cell type maturation, given the similarity between PC clusters and the CB clusters of the same glial type. A much more granular approach would be needed to properly analyze temporal changes in processes' mRNA composition, including obtaining larger datasets for the intermediate time points.

**Reviewer 3:**

The manuscript provides a comprehensive developmental transcriptomic atlas of Drosophila optic lobe glia by integrating scRNA datasets from larval to adult stages. The study is valuable resource for understanding glial diversity, developmental trajectories and neuron glia interactions. The identification of glial subtype transitions, particularly diversification of neuropil glia into astrocyte- like and ensheathing glia along with the discovery of process derived transcriptomic signatures, represents an important contribution. The combination of computational analysis and experimental validation strengthens the study. However, some points require clarification and additional support to full establish the proposed models:

1. Glial cell marker validation: provide some in vivo validation and comparison with established markers such as repo. Identification of new glial cell markers like CG32032, ANx19and GTE12 is valuable, however additional validation is needed to demonstrate their specificity across glial populations and developmental stages.

We agree with the reviewer on the relevance of properly validate *in vivo* the newly identified markers. In **Fig. 1F, G** and **Fig. S1E, F** we show in detail the expression in L3 and Adult optic lobes of the new CG32032 and AnxB9 antibodies that we had generated. We show that these new markers are expressed in glial cells labeled with Repo antibody. Interestingly, in the ventral nerve cord (VNC) dataset from Allen et al., 2020 (ref. 38), we found that *AnxB9* is strongly expressed in 4 non-glial clusters. Consistent with this, we found AnxB9 antibody in non-glial cells in the VNC (**Fig. S1I-H**).
 To assess antibody specificity, we have used UAS-RNAi constructs to knock down *AnxB9* and *CG32032* expression (**Fig. S7**). As discussed in the text: *"We were able to confirm the specificity of the AnxB9 antibody by RNAi knockdown, but could not do so for CG32032 antibody, probably due to inefficacy of the available RNAi construct (Fig. S7)."*

2. Mechanisms of glial diversification: Expand discussion of candidate regulatory pathways or transcriptional programs that may drive subtype specification. Address this issue in the discussion.

We appreciate the reviewer's point, and we have expanded our discussion to directly address the issue. We now highlight specifically the fact that we provide a molecular characterization of TFs that are expressed transiently at the point of ALG vs EG fate divergence (**Fig. 5E**) as well as the TFs differentially expressed along the pseudotime trajectories of ALG and EG mng (**Fig. S3E, E'**). On the other hand, we state directly that we have not functionally tested any candidate potentially involved in ALG vs EG specification.

3. Functional relevance of transcriptomic diversity: The atlas identify extensive transcriptional differences but their functional implications are not fully explored. The authors should discuss this issue.

This comment is related to the previous answer. The reviewer is correct, we have not directly addressed the mechanisms that drive ALG vs EG specification. However, we have explored this subject in the discussion: we present the results from Richier et al., 2017 (ref. 20), where they show that *Lapsyn* mutant ALG mng fail to arborize properly and acquire a morphology that resemble EG mng. We have not observed an ALG-specific expression of *lapsyn* or *htl*, but he have highlighted another candidate, the marker of ALG fate *wun2* (Lago-Baldaia et al., 2023, ref. 17) which has a clear ALG-specific expression. We have modified the Discussion to elaborate on this point and included a new supplementary Figure (**Fig. S8**) to show the expression of these relevant candidates found in the literature, and we have added a list of cell surface and secreted (CSS) genes differentially expressed over pseudotime to expand the potential candidates relevant to ALG vs. EG specification. We believe that the process of ALG vs EG fate acquisition is likely complex and would grant a dedicated study, and we expect that our molecular characterization of TF and CSS genes serve as a useful resource for the community.

4. Cell process-derived transcriptomic signatures: the identification of mRNA signature from glial process is innovative but additional validation is needed to distinguish biological localization from dissociation artifacts.

Given the novelty and relevance of our claim that the mechanical dissociation of large glial cells can generate fragments with and without nuclei than can subsequently be sequenced and annotated as PCs and CBs in single cell analyses, we have included strict controls in our manuscript to confirm our computational observations. In **Fig. S5C** we show that after the standard dissociation protocol for single cell transcriptomic experiments, there are nucleated and non-nucleated fragments of Chiasm glial cells (identified by Chiasm-Gal4 driven expression of membrane-bound GFP). On the other hand, in **Fig. 6Q** we use HCR-FISH to test whether the predictions we made for mRNA localization using our Chiasm_PC and Chiasm_CB annotation were valid. Specifically, we show that *repo* mRNA remains restricted to cell bodies (as predicted *in silico* in **Fig. 6F**), while *CG14598* mRNA is equally localized in projections and cell soma (see **Fig. 6M**). We consider that these experiments strongly support the biological validity of our transcriptional analysis.

This is a strong study that provides an important resource for the field. The findings are significant with some minor concerns. I recommend minor revision.
