# Supplementary material for: A developmental single-cell atlas of the Drosophila visual system glia reveals cell type diversification and subcellular mRNA compartmentalization
Source: PLoS Biol. 2026 Sep 28;24(9):e3004015. doi: 10.1371/journal.pbio.3004015 (PMC13649123; doi:10.1371/journal.pbio.3004015)
Supplement: Peer review history

# Editor report: Decision Letter 0

**DOI:** 10.1371/journal.pbio.3004015.r001

**Authors:** Ines Alvarez-Garcia

11 May 2026

Dear Claude,

Thank you for submitting your manuscript entitled "A Developmental Single-Cell Atlas of the Drosophila Visual System Glia Reveals Cell Type Diversification and Subcellular mRNA Compartmentalization" for consideration as a Research Article by PLOS Biology.

Your manuscript has now been evaluated by the PLOS Biology editorial staff as well as by an academic editor with relevant expertise and I am writing to let you know that we would like to send your submission out for external peer review.

However, before we can send your manuscript to reviewers, we need you to complete your submission by providing the metadata that is required for full assessment. To this end, please login to Editorial Manager where you will find the paper in the 'Submissions Needing Revisions' folder on your homepage. Please click 'Revise Submission' from the Action Links and complete all additional questions in the submission questionnaire.

Once your full submission is complete, your paper will undergo a series of checks in preparation for peer review. After your manuscript has passed the checks it will be sent out for review. To provide the metadata for your submission, please Login to Editorial Manager (https://www.editorialmanager.com/pbiology) within two working days, i.e. by May 13 2026 11:59PM.

If your manuscript has been previously peer-reviewed at another journal, PLOS Biology is willing to work with those reviews in order to avoid re-starting the process. Submission of the previous reviews is entirely optional and our ability to use them effectively will depend on the willingness of the previous journal to confirm the content of the reports and share the reviewer identities. Please note that we reserve the right to invite additional reviewers if we consider that additional/independent reviewers are needed, although we aim to avoid this as far as possible. In our experience, working with previous reviews does save time.

If you would like us to consider previous reviewer reports, please edit your cover letter to let us know and include the name of the journal where the work was previously considered and the manuscript ID it was given. In addition, please upload a response to the reviews as a 'Prior Peer Review' file type, which should include the reports in full and a point-by-point reply detailing how you have or plan to address the reviewers' concerns.

During the process of completing your manuscript submission, you will be invited to opt-in to posting your pre-review manuscript as a bioRxiv preprint. Visit http://journals.plos.org/plosbiology/s/preprints for full details. If you consent to posting your current manuscript as a preprint, please upload a single Preprint PDF.

Feel free to email us at plosbiology@plos.org if you have any queries relating to your submission.

Kind regards,

Ines

--

Ines Alvarez-Garcia, PhD

Senior Editor

PLOS Biology

ialvarez-garcia@plos.org

# Peer review: Decision Letter 1

**DOI:** 10.1371/journal.pbio.3004015.r002

**Authors:** Ines Alvarez-Garcia

13 Jul 2026

Dear Claude,

Thank you for your patience while your manuscript entitled "A Developmental Single-Cell Atlas of the Drosophila Visual System Glia Reveals Cell Type Diversification and Subcellular mRNA Compartmentalization" went through peer-review at PLOS Biology, and please accept my apologies for the delay. Your manuscript has now been evaluated by the PLOS Biology editors, an Academic Editor with relevant expertise, and by three independent reviewers.

The reviews are attached below. As you will see, the reviewers find the study interesting and worth pursuing, but they also raise several issues that would need to be addressed. Reviewer 1 asks for several clarifications and a further discussion on whether or not similar phenomena of glia fate diversification and plasticity exist in other systems or model organisms. Reviewer 2 thinks that a scheme of the fly glia should be added for non-experts and also that some discrepancies of the results with other papers should be discussed. Finally, Reviewer 3 thinks the glia markers identified should be validated in vivo to demonstrate their specificity and also the mRNA signature from glial process to distinguish biological localization from dissociation artifacts.

In light of the reviews, we are pleased to offer you the opportunity to address the comments from the reviewers in a revision that we anticipate should not take you very long. In addition, we would like to consider your manuscript as a 'Method and Resources' type of article, thus please select that type of article from the drop-down menu when you resubmit. We will then assess your revised manuscript and your response to the reviewers' comments with our Academic Editor aiming to avoid further rounds of peer-review, although we might need to consult with the reviewers, depending on the nature of the revisions.

Please also address the data and other policy-related requests stated below my signature.

In addition to these revisions, you may need to complete some formatting changes, which you will receive in a follow up email. A member of our team will be in touch with a set of requests shortly. If you do not receive a separate email within a few days, please assume that checks have been completed, and no additional changes are required.

We expect to receive your revised manuscript within 1 month. Please email us (plosbiology@plos.org) if you have any questions or concerns, or would like to request an extension.

At this stage, your manuscript remains formally under active consideration at our journal; please notify us by email if you do not intend to submit a revision so that we withdraw the manuscript.

**IMPORTANT - SUBMITTING YOUR REVISION**

Your revisions should address the specific points made by each reviewer. Please submit the following files along with your revised manuscript:

1. A 'Response to Reviewers' file - this should detail your responses to the editorial requests, present a point-by-point response to all of the reviewers' comments, and indicate the changes made to the manuscript.

*NOTE: In your point-by-point response to the reviewers, please provide the full context of each review. Do not selectively quote paragraphs or sentences to reply to. The entire set of reviewer comments should be present in full and each specific point should be responded to individually.

You should also cite any additional relevant literature that has been published since the original submission and mention any additional citations in your response.

2. In addition to a clean copy of the manuscript, please also upload a 'track-changes' version of your manuscript that specifies the edits made. This should be uploaded as a "Revised Article with Changes Highlighted " file type.

3. Resubmission Checklist

When you are ready to resubmit your revised manuscript, please refer to this resubmission checklist: https://plos.io/Biology_Checklist

To submit a revised version of your manuscript, please go to https://www.editorialmanager.com/pbiology/ and log in as an Author. Click the link labelled 'Submissions Needing Revision' where you will find your submission record.

*Published Peer Review*

Please note while forming your response, if your article is accepted, you may have the opportunity to make the peer review history publicly available. The record will include editor decision letters (with reviews) and your responses to reviewer comments. If eligible, we will contact you to opt in or out. Please see here for more details:

https://blogs.plos.org/plos/2019/05/plos-journals-now-open-for-published-peer-review/

Thank you again for your submission to our journal. We hope that our editorial process has been constructive thus far, and we welcome your feedback at any time. Please don't hesitate to contact us if you have any questions or comments.

Sincerely,

Ines

--

Ines Alvarez-Garcia, PhD

Senior Editor

PLOS Biology

ialvarez-garcia@plos.org

------------------------------------------------------------------------

DATA POLICY:

You may be aware of the PLOS Data Policy, which requires that all data be made available without restriction: http://journals.plos.org/plosbiology/s/data-availability. For more information, please also see this editorial: http://dx.doi.org/10.1371/journal.pbio.1001797

Note that we do not require all raw data. Rather, we ask that all individual quantitative observations that underlie the data summarized in the figures and results of your paper be made available in one of the following forms:

1) Supplementary files (e.g., excel). Please ensure that all data files are uploaded as 'Supporting Information' and are invariably referred to (in the manuscript, figure legends, and the Description field when uploading your files) using the following format verbatim: S1 Data, S2 Data, etc. Multiple panels of a single or even several figures can be included as multiple sheets in one excel file that is saved using exactly the following convention: S1_Data.xlsx (using an underscore).

2) Deposition in a publicly available repository. Please also provide the accession code or a reviewer link so that we may view your data before publication.

Regardless of the method selected, please ensure that you provide the individual numerical values that underlie the summary data displayed in the following figure panels as they are essential for readers to assess your analysis and to reproduce it:

Fig. 1C, D; Fig. 2A, B; Fig. 3A, B; Fig. 4A; Fig. 5A-E; Fig. 6D-O; Fig. S1A, A’, C; Fig. S2A, B, E, F; Fig. S3A-E’; Fig. S4A-I; Fig. S5A, B and Fig. S6B-E

NOTE: the numerical data provided should include all replicates AND the way in which the plotted mean and errors were derived (it should not present only the mean/average values).

Please also ensure that figure legends in your manuscript include information on WHERE THE UNDERLYING DATA CAN BE FOUND, and ensure your supplemental data file/s has a legend.

Please ensure that your Data Statement in the submission system accurately describes where your data can be found.

------------------------------------------------------------------------

CODE POLICY

Per journal policy, if you have generated any custom code during the course of this investigation, please make it available without restrictions. Please ensure that the code is sufficiently well documented and reusable, and that your Data Statement in the Editorial Manager submission system accurately describes where your code can be found. More information on our Code Policy, what and how to share can be found here: https://journals.plos.org/plosbiology/s/code-availability

Please note that we cannot accept sole deposition of code in GitHub, as this could be changed after publication. However, you can archive this version of your publicly available GitHub code to Zenodo. Once you do this, it will generate a DOI number, which you will need to provide in the Data Accessibility Statement (you are welcome to also provide the GitHub access information). See the process for doing this here: https://docs.github.com/en/repositories/archiving-a-github-repository/referencing-and-citing-content

------------------------------------------------------------------------

DATA NOT SHOWN or UNPUBLISHED

- Please note that per journal policy, we do not allow the mention of "data not shown/unpublished", "personal communication", "manuscript in preparation" or other references to data that is not publicly available or contained within this manuscript. Please either remove mention of these data or provide figures presenting the results and the data underlying the figure(s).

----------------------------------------------------------------

Reviewers' comments

Rev. 1:

This well-documented scRNA-seq analysis of glia diversity through fly optic lobe development by the Claude lab yields novel insights on fundamental developmental biology. It establishes the fly optic lobe as a supreme model system for deep mechanistic studies on neuron-glia interactions in neural network formation, function, and plasticity.

Trajectory analysis of scRNA-seq from late larval to mid pupal stages is informative and central to this study. The observation that a single pool of neuropil glia diversifies into ensheathing and astrocyte-like glia during network formation is intriguing. The discussions on three possible underlying mechanisms are insightful.

Minor points to be clarified include:

1. About the origin of medulla neuropil glia, is it known how many neuropil glia are made from a single medulla neuroblast? If more than one, how about the glia diversity from a single medulla neuroblast?

2. It is unclear about the nature of lineage tracing on Hand-expressing medulla neuropil glia. Any evidence of cell proliferation?

3. Please comment if similar phenomena of glia fate diversification and plasticity are observed in other systems and model organisms.

Rev. 2:

This is an impressive and important study that leverages existing scRNAseq datasets to delineate optic lobe glial developmental trajectories, subtypes, and intriguing subcellular differences between soma and processes. The analyses here will open new avenues for studying and manipulating fly glia, as well as for separating soma from cellular processes in scRNAseq datasets.

I support publication of this study with no additional experiments required, but with some minor and major suggestions to make the MS more broadly readable outside of the fly field.

Major comments:

1) Fig1: before diving into approach, a schematic of the fly glia here (even if similar to take-home messages in Fig7) would be very useful for non-experts

2) Fig6 is REALLY important. I would highly encourage authors to put schema of the problem that they identified (cell body vs processes) at top of figure. Authors are encouraged to show top 10 DEGs for PC vs CB, for both cortex glia and chiasm glia? (or show GO analysis, but DEGs maybe more useful). I am wondering if the graphed gene examples (ie fabp) are the most differentially expressed, or were selected for other reasons such as historical precedent for process enrichment

3) Do the authors have any examples of interpretations that were changed upon excluding processes compared to soma? Are developmental trajectories altered by the inclusion of these processes? (is important information contained exclusively in these processes that is lost if those clusters are excluded from the bulk of the analyses in this data?).

4) The authors propose a pipeline for identifying CB vs PC. Does this work in non-fly or different labs' data? Attempting the pipeline on other organism, or commenting on if this problem exists in other datasets, would be greatly appreciated by the broader community! Would a similar problem exist for neuronal soma vs processes?

a. For example, in PMID 38839958, FigS1f, elav and nSyb reads appear quite high in repo+ glia sorted by FACS from repo-GAL4 > TRE-dsRed,UAS-GFP brains. Author logic is that neurons are dsRednegGFPneg, AP1neg glia are dsRednegGFP + , and AP1+ glia are dsRed+GFP +. Wondering if the soma vs PC is driving some of this messiness.

5) Kato et al (PMID 32051172) claim that "larval EG dedifferentiate, proliferate and redifferentiate into adult ALG and EG.", based on analysis with Gs2+ and Naz+ IHC experiments in the central brain. Similarities between ALG and EG subtypes is also suggested by this paper, with "neuropil glia that transcriptionally appears as a single cell class in larvae, splits into ensheathing (EG) and astrocyte-like (ALG) glia types during pupal stages." If possible, authors may comment on these different outcomes (noting also optic lobe versus central brain differences) in the discussion.

Minor comments:

1) "Cortex glia is" and similar examples of pluralities of glia given singular verbs exist in the MS; I encourage authors to make sure collective groupings of glia are given plural verbs!

a. Ie "neuropil glia that transcriptionally appears as a single cell class in larvae, splits into ensheathing (EG) and astrocyte-like (ALG) glia types during pupal stages."

2) Digestibility of all figures would be improved by having the life stage noted within the figure panel rather than only fig legend (ie Fig. 1D as L3 and Fig. 1E as adult).

3) Authors mention that mbp translation is induced by activity; is there any relationship between electrically active brains (>P50) and the types or abundances of cortex glial process transcriptomes?

Rev. 3:

The manuscript provides a comprehensive developmental transcriptomic atlas of Drosophila optic lobe glia by integrating scRNA datasets from larval to adult stages. The study is valuable resource for understanding glial diversity, developmental trajectories and neuron glia interactions. The identification of glial subtype transitions, particularly diversification of neuropil glia into astrocyte- like and ensheathing glia along with the discovery of process derived transcriptomic signatures, represents an important contribution. The combination of computational analysis and experimental validation strengthens the study. However, some points require clarification and additional support to full establish the proposed models:

1. Glial cell marker validation: provide some in vivo validation and comparison with established markers such as repo. Identification of new glial cell markers like CG32032, ANx19and GTE12 is valuable, however additional validation is needed to demonstrate their specificity across glial populations and developmental stages.

2. Mechanisms of glial diversification: Expand discussion of candidate regulatory pathways or transcriptional programs that may drive subtype specification. Address this issue in the discussion.

3. Functional relevance of transcriptonic diversity: The atlas identify extensive transcriptional differences but their functional implications are not fully explored. The authors should discuss this issue.

4. Cell process-derived transcriptomic signatures: the identification of mRNA signature from glial process is innovative but additional validation is needed to distinguish biological localization from dissociation artifacts.

This is a strong study that provides an important resource for the field. The findings are significant with some minor concerns. I recommend minor revision.

# Author comment: Author response to Decision Letter 2

**DOI:** 10.1371/journal.pbio.3004015.r003

13 Aug 2026

Attachment Submitted filename: Response to Reviewers_08.11.2026.docx

# Editor report: Decision Letter 2

**DOI:** 10.1371/journal.pbio.3004015.r004

**Authors:** Ines Alvarez-Garcia

10 Sep 2026

Dear Claude,

Thank you for the submission of your revised Methods and Resources entitled "A Developmental Single-Cell Atlas of the Drosophila Visual System Glia Reveals Cell Type Diversification and Subcellular mRNA Compartmentalization" for publication in PLOS Biology. On behalf of my colleagues and the Academic Editor, Bing Ye, I am delighted to let you know that we can in principle accept your manuscript for publication, provided you address any remaining formatting and reporting issues. These will be detailed in an email you should receive within 2-3 business days from our colleagues in the journal operations team; no action is required from you until then. Please note that we will not be able to formally accept your manuscript and schedule it for publication until you have completed any requested changes.

Please take a minute to log into Editorial Manager at http://www.editorialmanager.com/pbiology/, click the "Update My Information" link at the top of the page, and update your user information to ensure an efficient production process.

PRESS

We frequently collaborate with press offices. If your institution or institutions have a press office, please notify them about your upcoming paper at this point, to enable them to help maximise its impact. If the press office is planning to promote your findings, we would be grateful if they could coordinate with biologypress@plos.org. If you have previously opted in to the early version process, we ask that you notify us immediately of any press plans so that we may opt out on your behalf.

We also ask that you take this opportunity to read our Embargo Policy regarding the discussion, promotion and media coverage of work that is yet to be published by PLOS. As your manuscript is not yet published, it is bound by the conditions of our Embargo Policy. Please be aware that this policy is in place both to ensure that any press coverage of your article is fully substantiated and to provide a direct link between such coverage and the published work. For full details of our Embargo Policy, please visit http://www.plos.org/about/media-inquiries/embargo-policy/.

Many congratulations and thanks again for choosing PLOS Biology for publication and supporting Open Access publishing. We look forward to publishing your study.

Sincerely,

Ines

--

Ines Alvarez-Garcia, PhD

Senior Editor

PLOS Biology

ialvarez-garcia@plos.org
